# Fabp4, a new player in the adipo-pancreatic axis

**DOI:** 10.1016/j.molmet.2014.03.009

**Published:** 2014-04-03

**Authors:** Risheng Ye, Philipp E. Scherer

**Affiliations:** 1 Touchstone Diabetes Center, Department of Internal Medicine, University of Texas Southwestern Medical Center, Dallas, TX, USA; 2 Department of Cell Biology, University of Texas Southwestern Medical Center, Dallas, TX, USA

As an early hallmark of type 2 diabetes, the associated insulin resistance in peripheral tissues demands extra insulin production from the pancreatic β cells. Circulating glucose has long been recognized as the primary messenger, triggering acute and prolonged insulin secretion via well-defined biochemical pathways in β cells. However, additional mechanisms are in place to safeguard against lagging islet mass and function. Recent efforts have cast light on the crosstalk between peripheral tissues and β cells, mediated by specific protein factors. Skeletal muscle, in the absence of PGC-1α, releases excessive levels of pro-inflammatory IL-6 to blunt the glucose-stimulated insulin secretion (GSIS) from islets [1]. Others report that upon exposure to TNF-α, insulin-resistant myocytes release a panel of cytokines that impair GSIS, while normal muscle cells produce hormones that are beneficial to β cell function and proliferation [2]. As for factors derived from the liver and adipose tissue, Angptl8 (a.k.a. betatrophin) has been implicated as a possible stimulator of β cell proliferation [3], though the relevance of this finding for human islet proliferation has recently been questioned [4].

In this issue of Molecular Metabolism, Cantley and colleagues report a novel role of the adipocyte-specific fatty acid-binding protein Fabp4 (aP2) in mediating β cell GSIS [5]. This group set out to test the hypothesis that upon obesity and hypoxia, adipose tissues convey signals to pancreatic β cells to indicate the need for enhanced insulin production. This adipocyte-derived signal would account for, at least in part, the hyperinsulinemia observed during insulin resistance. With mass spectrometry analysis of the proteins in the conditioned medium of 3T3-L1 adipocytes, the authors identified a small number of proteins that were both secreted and upregulated under hypoxic conditions. Sparcl1 and the α1 subunit of collagen VI were amongst them. Both of these are extracellular matrix proteins, presumably active only in the local microenvironment and seemingly ill-suited as an endocrine signal. Future studies will have to address whether these factors or cleavage products of these proteins may serve as signals at the systemic level. A lower-hanging fruit amongst these hypoxia-induced proteins was Fabp4. Even though conventionally thought of as an intracellular, cytoplasmic protein, there is considerable momentum for the idea that Fabp4 has the potential to be a secreted molecule as well, even though its cellular release is not mediated through the conventional secretory pathway. Fabp4 had previously been implicated as a potent stimulator of β3-adrenergic agonist action, leading to stimulated acute insulin secretion [6]. This suggested that Fabp4 may serve as a signaling molecule from the adipocyte to the β cell. Indeed, β3-adrenergic agonist stimulation prompts an increase in Fabp4 release from the adipocyte. Additional experiments, both *in vitro* and *in vivo*, were performed to demonstrate that Fabp4 affects GSIS in β cells. Upon continued elevation of Fabp4 in circulation (using minipump implants containing recombinant Fabp4), the authors observe enhanced GSIS. Correlational data from clinical samples reveals a situation consistent with the model proposed in rodents. Serum Fabp4 levels are elevated in subjects with elevated body mass index (BMI), and these circulating Fabp4 levels are in turn directly proportional to GSIS. Both in rodents and in humans, insulin suppresses the further release of Fabp4 from adipocytes, indicative of a feedback response that prevents excessive insulin release.

It is an intuitively attractive model to have the adipocyte control its response to actual or perceived insulin resistance by increasing the release of a messenger molecule, whose secretion triggers an enhanced output of insulin, which in turn feeds back onto the adipocyte to suppress the insulin secretagogue release. It may not be local insulin sensitivity per se that is the direct driving force for enhanced Fabp4 release. As Cantley and colleagues emphasize in this report, hypoxia arises from inadequate vasculature in rapidly extending fat. Hypoxia can lead to fibrosis and insulin resistance as a secondary event, via induction of HIF1α [7]. Adipose tissue expansion is not limited to adipocyte hyperplasia and hypertrophy, but tightly integrated with angiogenesis, inflammatory responses and extracellular matrix (ECM) deposition [8]. Fibrosis modulates angiogenesis and macrophage infiltration, and is linked to adipose dysfunction [9]. This complex state of adipocyte dysfunction may be what drives Fabp4 release.

Many questions remain to be answered: How is a seemingly tightly regulated secretory response achieved with a protein that does not depend on the classical secretory pathway? How can insulin suppress this process so rapidly? How is the Fabp4 response physiologically integrated with the action of the large number of other known insulin secretagogues, such as the components of the GLP-1 axis? Finally, how does Fabp4 achieve its effects on the β cell?

This is further evidence that there are a number of additional unsuspected functions for this otherwise rather inconspicuous intracellular lipid binding protein, beyond its previously assigned systemic role [10]. It will be exciting to hear about the next chapter of Fabp4, as a ligand for a receptor or as a carrier for a bioactive lipid as its cargo, triggering biological responses at the level of target cells.
